# Barriers to appropriate complementary feeding and the use of ultra‐processed foods: A formative qualitative study from rural Oromia, Ethiopia

**DOI:** 10.1111/mcn.13576

**Published:** 2023-12-04

**Authors:** Elazar Tadesse, Ibrahim Abdirahman, Shiferaw Letta, Miles Kirby, Tigist Mamo, Henok Metaferia, Beryl Oranga, Jessica Leight

**Affiliations:** ^1^ Nutrition Department Menelik II Medical and Health Science College Addis Ababa Ethiopia; ^2^ Dunamis Consultancy Addis Ababa Ethiopia; ^3^ Department of Women's and Children Uppsala University Uppsala Sweden; ^4^ Department of Social Work, Faculty of Social Sciences Arsi University Asela Ethiopia; ^5^ Department of Nursing, College of Health Science and Medicine Haramaya University Harar Ethiopia; ^6^ World Vision Washington D.C. USA; ^7^ World Vision Addis Ababa Ethiopia; ^8^ Poverty, Gender and Inclusion Unit International Food Policy Research Institute Washington District of Columbia USA

**Keywords:** complementary feeding, Ethiopia, infant and young child feeding practices, ultra‐processed foods

## Abstract

Children's consumption of ultra‐processed foods (UPF) is increasing in Ethiopia, but relatively little is known about the specific feeding practices that underlie this pattern. The objective of this study was to explore patterns of consumption of UPF by infants and young children within a broader context of inappropriate complementary feeding practices in extremely poor households in rural Oromia, Eastern Ethiopia. A formative qualitative study was conducted using semistructured interview questionnaires developed drawing on a socioecological model. A total of 16 focus group discussions with mothers (45 respondents), fathers (21 respondents) and grandmothers (23 respondents) of children aged 6–23 months in households that were beneficiaries of the Productive Safety Net Program were conducted, along with four key informant interviews with health workers. Qualitative transcripts were complemented with field notes before qualitative content analysis was applied. The key findings suggest that UPF were widely provided to infants and young children as part of a pattern of suboptimal complementary feeding, including both early and late initiation of complementary foods. In particular, UPF (including juice, biscuits and lipid‐based nutrient supplements) were diluted with or dissolved in water and fed to infants via bottle, often before the recommended age of initiation of 6 months. Mothers and caregivers reported that they perceived the products to be affordably priced and packaged, ready to use and convenient given their time constraints. The level of consumption of UPF and its effects on infant and young child feeding feeding practices and children's nutritional status in rural Ethiopia should be further explored.

## Key messages

- A range of inappropriate complementary feeding practices for infants and young children were observed in this sample of extremely poor households in rural Oromia, including both early and late initiation of complementary foods and an overreliance on a liquid diet.
- The perception that young children should initiate complementary feeding with liquids resulted in the heavy use of ultra‐processed foods (UPF), such as biscuits and packed juice, soft drinks and lipid‐based nutrient supplements; these foods were reported to be dissolved in or diluted with water.
- Respondents reported that UPF were available for purchase in small and affordable quantities, and also convenient to provide given maternal time constraints.
- Provision of UPF may increase the risk of gastrointestinal illness due to exposure to unclean water, and may also increase the risk of a nutritionally inadequate diet.

## Graphical abstract

This qualitative analysis explored patterns of complementary feeding and consumption of ultra‐processed foods by infants and young children in poor rural households that are beneficiaries of the Productive Safety Net Program in Ethiopia. The evidence shows that these foods are often introduced during the period of complementary feeding because mothers and caregivers believe them to be nutritious, convenient (when diluted and provided to infants via bottle) and affordably priced.

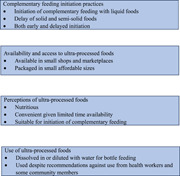

## 1 INTRODUCTION

Poor complementary feeding practices, including the untimely initiation of complementary foods and the introduction of inappropriate foods such as ultra‐processed foods (UPF), are increasingly major contributors to the poor nutritional status of children in lower and middle‐income countries. We follow recent literature to define UPF as foods manufactured using industrial formulations and a large number of ingredients, including a range of additives typically employed only in manufactured foods (Gibney, 2019). Children's consumption of unhealthy foods has been identified as a new threat affecting children's health globally (World Health Organization, 2020). The rising prevalence of this phenomenon was also reflected in the recent revision of World Health Organization infant and young child feeding (IYCF) guidelines to capture unhealthy feeding practices, along with the traditional focus on the nutritional adequacy of complementary foods (WHO and UNICEF, 2021). Infants and young children require a minimum recommended diet to ensure appropriate growth and development, and appropriate complementary feeding can reduce the risk of poor nutrition and enhance development (Dewey & Vitta, 2013).

Evidence around patterns of consumption of UPF for young children in lower and middle‐income countries is limited. One recent quantitative study evaluated commercial baby food availability in Natal, Brazil, and demonstrated that 79% of foods available for sale and targeted at children under 36 months were UPF (da Rocha et al., 2021). The prevalence of consumption of commercial snacks and beverage items was also identified as high among children in four countries in West Africa (Nordhagen et al., 2019). In rural Ethiopia, a recent study demonstrated that UPF characterised by low nutritional value are increasingly consumed by children, despite a persistent pattern of low dietary diversity (Tizazu et al., 2022). Understanding more about how patterns of consumption of UPF relate to broader patterns of complementary feeding and how UPF are perceived and used by caregivers is important for the design of effective nutrition education messages.

The objective of this formative qualitative study was to explore the evidence around young children's consumption of UPF as part of a broader pattern of inappropriate complementary feeding practices within a sample of extremely poor households (all beneficiaries of the Productive Safety Net Program [PSNP]) in rural Oromia, Eastern Ethiopia.

## 2 METHODS

### 2.1 Study setting and objectives

This analysis draws on formative qualitative work conducted during the first year of SPIR II, the Strengthen Productive Safety Net Program Institutions and Resilience Resilience Food Security Activity (RFSA). SPIR II seeks to sustain nutrition security, reduce risks to livelihoods and strengthen social safety nets while building on the PSNP, one of the largest social safety net programmes in sub‐Saharan Africa. The PSNP provides food or cash transfers targeted to poor households in the form of payments for seasonal labour on public works or as direct support to households and has been shown to reduce household food insecurity and increase asset holdings (Berhane, 2014; Hoddinott et al., 2012). Funded by USAID's Bureau for Humanitarian Assistance (BHA) and in close collaboration with the Government of Ethiopia, World Vision leads the implementation of the SPIR RFSA, in partnership with the Organization for Rehabilitation and Development in Amhara (ORDA) and CARE.

This formative qualitative work was conducted as part of the SPIR II learning agenda led by the International Food Policy Research Institute (IFPRI). The global research question can be described as follows: what key factors drive the adoption of inappropriate complementary feeding practices for infants and young children among PSNP beneficiary households (generally the poorest 10%–15% of rural households) in Ethiopia? Key secondary research questions included the following: what are the challenges linked to available financial resources that lead to the late introduction of complementary foods? How important are challenges linked to the limited availability of nutritious foods and/or higher availability of unhealthy or highly processed foods? Are constraints on maternal time or limited knowledge or engagement in active or responsive feeding salient in shaping feeding practices? What role is played by limited knowledge around the appropriate timing and methods of introduction of complementary foods, and over‐adherence to exclusive breastfeeding?

Data collection was conducted from April 27 to May 12, 2022, in East and West Hararge zones, Oromia, Eastern Ethiopia. Our objective was to collect data from PSNP households in a region served by SPIR II, operating in Amhara and Oromia; given that ongoing insecurity rendered many subregions of Amhara impassable, data collection was conducted in Oromia only, and zones were selected based on accessibility to the research team. Within these zones, four kebeles (the smallest administrative unit in Ethiopia) were purposively selected. The kebeles were selected based on the high and low prevalence of on‐time introduction of complementary foods as reported by mothers of infants 6–12 months, measured in a previous large‐scale survey conducted in the region in 2021 (Alderman et al., 2021). Based on this data, the lowest prevalence of on‐time introduction of complementary foods was 25% in Melkayee kebele, followed by 60% at Haqa Bas, 83.3% at Kurfa Challe and 100% in Kortu; however, these estimates were based on relatively small samples of mother–infant dyads observed in the quantitative survey (usually, no more than 10 infants of the target age, 6–12 months, were observed in each kebele).

### 2.2 Conceptual framework

The conceptual framework for this study was provided by Bronfenbrenner's socioecological model, conceptualising the relationships within the diverse environments surrounding children, including the physical, mental and social contexts for health and health interventions (Bronfenbrenner, 1994). The model was used to explore complementary feeding practices and the underlying reasons for those practices. This model is believed to be most relevant to nutrition social behavioural change communication and has been adopted in the formulation of the multi‐sectoral nutrition strategy of organisations, such as USAID (2014).

In this study, the model components formed the basis for the development of semistructured guides for data collection. The key topical areas for data collection included patterns of feeding practices for children under 24 months of age and particularly under 12 months of age; individual, household and community‐level barriers to appropriate feeding practices; preferred complementary foods (including UPF) and strategies employed to choose the timing of introduction of complementary foods; and resources available to support optimal complementary feeding practices. While the investigation encompassed a wider range of questions linked to complementary feeding practices described above in key research questions, this analysis focuses particularly on reported patterns of consumption of UPF (including household's access to and use of UPF, and their perceptions of UPF) given the evidence that emerged in data collection that this was a particularly important challenge in this context. Further papers may explore other topics included in the investigation.

### 2.3 Participants and sample size

Participants in the qualitative data collection included mothers (*n* = 45 respondents in eight focus groups), fathers (*n* = 21 respondents in four focus groups), grandmothers (*n* = 23 in four focus groups) and health workers (*n* = 4 in four key informant interviews). All participants were purposively selected and recruited based on convenience and eligibility. The eligibility criteria for mothers and fathers were as follows: all respondents were characterised by the presence of at least one child 6–23 months of age in the household, and all households were currently participating in the PSNP. For grandmothers, the same criteria applied for the household in which the child resided, but it was not required that the grandmother be coresident with the child. (Mothers, fathers and grandmothers were all from different families.) For health workers, we sampled health extension workers (HEWs); three of the four kebeles had only one HEW present (the second HEW position was vacant), and in one kebele (Kortu) characterised by the presence of two HEWs, the HEW identified as the head of the health post was interviewed.

The mothers were further categorised into two groups: those characterised by a parity higher than two were considered experienced mothers for the purposes of data collection (*n* = 23), while those characterised by parity one or two were considered inexperienced mothers (*n* = 22). Our goal in sampling experienced mothers was to ensure that the data included a sufficient number of mothers with multiple (and thus older) children, who had cared for a child through the full transition from exclusive breastfeeding to exclusive consumption of solid food (from approximately 6 to 36 months). This may have provided them with unique insights into the evolution of feeding practices over a longer period. As the target samples for fathers and grandmothers were smaller, these respondents were not differentiated based on child (or grandchild) parity.

Respondents were identified by local staff of nongovernmental organisations who deliver the PSNP and were sampled based on convenience and proximity to the site for focus group discussions (FGD). All respondents invited (*n* = 93) agreed to participate.

### 2.4 Data collection

Data were collected using 16 FGD and four key informant interviews (KII). Within each of the four kebeles, we conducted four FGDs per kebele: (1) mothers of parity one or two currently caring for a child aged 6–23 months (inexperienced mothers); (2) mothers of parity three or higher currently caring for a child in the same age range (experienced mothers); (3) fathers with at least one child in the same age range; (4) grandmothers with at least one grandchild in the same age range. (Note that fathers and mothers are required to be currently coresiding with a child in the target age range.) Thus the total sample included 16 FGDs. A KII was also conducted with one HEW in each selected kebele for a total of four KIIs.

All FGDs and KIIs were conducted in the Afaan Oromo language using semistructured interview guides. Each focus group consisted of five to six participants and lasted 48–90 min, with an average duration of 53.5 min. For the four KIIs conducted, the duration ranged between 27 and 48 min, with an average duration of 35.4 min.

The research team included a facilitator who led the interviews (E. T.), a note‐taker and a field supervisor. The facilitator and note‐taker (I. A. and S. L.) are based at universities in the Oromia region and are native speakers of Afaan Oromo, with experience working on qualitative research in the region. The field supervisor was the principal investigator (E. T.), who has an intermediate mastery of the Afaan Oromo language. All interviews were audio‐recorded after obtaining informed verbal consent, and extensive field notes were taken by the note‐taker (one of the two Oromia‐based researchers noted above) during each interview. Almost all FGDs were conducted in the morning since the data collection was during the Ramadan fasting period, and participants were unable to attend discussions in the afternoon; as noted below, the sample population here is predominantly Muslim. Accordingly, field notes were typed in detail in English daily following the focus groups. When field notes lacked clarity, the audio records were played to refine the field notes, and the recordings were also used to identify illustrative quotes. To assist in future analysis, all field notes were organised under the main themes in the interview guides. Topics that emerged in the discussion that were not part of the interview guide were also included in the field notes under the most closely related theme.

### 2.5 Data analysis

Following the finalisation of field data collection, audio recordings were transcribed into Afan Oromo and translated into English. The field notes from each interview were added to the respective translated data and the whole document was treated as a unit of analysis and imported to MAXQDA software. Thereafter, inductive analysis was conducted in which first meaning units—that is, a constellation of words or statements that relate to the same central meaning—were identified and summarised. Second, the identified meaning units were coded, and third, subcategories were developed based on similarities and differences in content and codes. Finally, categories that link the underlying meanings together were developed (Graneheim & Lundman, 2004).

An example of the process of analysis is found in Table 1. A series of meaning units related to the initiation of complementary feeding, the timing of the introduction of various foods and methods of feeding are identified. Similar meaning units identified in multiple focus groups are assigned parallel codes. The coded concepts are then grouped into subcategories based on the interrelationships between these concepts, and subcategories are similarly grouped into four large categories to structure the analysis. These four categories correspond to the major themes that dominated the discussions: complementary feeding initiation practices, access to UPF, perceptions of UPF, and use of UPF.

**Table 1:** An example of the process of data analysis.

Meaning units	Code	Subcategories	Categories
We start feeding them at 6 months. We prepare a thinned soup … Make them licking by their tongue. We put it in to their mouth. They can't eat solid foods.	Complementary food	Initiated with liquid foods	Complementary feeding initiation practices
Before a year, solid food are not allowed to feed the baby, therefore it must be after a year … . You should not give large amount.	Age for giving solid/semisolid foods	Delay in the introduction of solid and semisolid foods	
… from the birth to 6 months … breast milk; after that we start to provide foods … we boil milk and give the child; from shops we buy juice and dilute and feed …	Diluted ultra‐processed food	Dissolved in or diluted with water	How ultra‐processed foods are used
my child is not satisfied with the mother's breast milk … when she cries I dilute barley and sugar and feed her using bottle.	Method of feeding	Bottle feeding	
	Sugar	Added to thin porridge as sweeter	

### 2.6 Ethics statement

Ethical approval was obtained from the Institutional Review Boards of the Ethiopian Public Health Association (EPHA/06/815/22) and IFPRI (PHND‐22‐0420). In addition, permission was obtained from relevant federal and local government offices. Informed verbal consent was obtained from all participants and documented through audio recording before the start of the interview. In addition, the research team maintained all appropriate COVID‐19 protocols. The research team used proper face coverings in any interaction with respondents and offered masks to all participants.

## 3 RESULTS

### 3.1 Sociodemographic characteristics of study participants

Table 2 describes the characteristics of the study participants. The mean (standard deviation [SD]) of age reported by mothers, fathers and grandmothers was 25.1 (5.7), 34.3 (11.1) and 45.6 (12.5) years, respectively. About 70% of the respondents report two or more under‐5 year children in their households. The index child is identified as the youngest child in the household of the respondent; 51.7% of index children were girls, and the mean (SD) age of the index child was 13.8 (5.8) months. Data on participant religion was not collected, but the region is predominantly Muslim (Edossa et al., 2021).

**Table 2:** Background characteristics of mothers, fathers and grandmothers of children 6–23 months of age (*n* = 89).

Characteristics	Pooled sample (*n* = 89)	Mothers (*n* = 45)	Fathers (*n* = 21)	Grandmothers (*n* = 23)
Age in years				
Mean (SD)	33.1 (13.1)	25.1 (5.7)	34.3 (11.1)	45.6 (12.5)
Number of under‐five children in household, *N* (%)				
One	27 (30.3)	16 (35.5)	5 (23.8)	6 (26.1)
Two	37 (41.6)	17 (37.8)	13 (61.9)	7 (30.4)
Three or more	25 (28.1)	12 (26.7)	3 (14.4)	10 (43.5)
Age of index child in months				
Mean (SD)	13.8 (5.8)	14.1 (5.6)	12.8 (6.0)	14.2 (5.8)
Sex of index child, *N* (%)				
Boy	43 (48.3)	21 (46.7)	11 (52.3)	11 (47.8)
Girl	46 (51.7)	24 (53.3)	10 (47.7)	12 (52.2)

### 3.2 Overview of qualitative coding

The qualitative analysis identified four categories relevant to understanding complementary feeding practices and their relationship to the use of UPF, as summarised in Table 3.

**Table 3:** Categories and subcategories identified during the analysis.

Complementary feeding initiation practices
Initiation of complementary feeding with liquid foods Delay of solid and semi‐solid foods Both early and delayed initiation
Availability and access to ultra‐processed foods
Available in small shops and marketplaces Packaged in small affordable sizes Lipid‐based nutrient supplements (LNS, branded internationally as Plumpy Nut) are available in shops and marketplaces (despite its designation for use to treat acute malnutrition at health facilities)
Perceptions of ultra‐processed foods
Nutritious Convenient given the limited‐time availability Suitable for initiation of complementary feeding Effective in preventing malnutrition
Use of ultra‐processed foods
Dissolved in or diluted with water for bottle feeding Added to other foods (e.g., thin porridge) as a sweetener Used despite recommendations against use from health workers and some community members

### 3.3 Complementary feeding initiation practices

Upon initiation of complementary feeding, almost all of the mothers and grandmothers in the study sample reported providing young children with a liquid diet until approximately 9–12 months of age. Diluted cow or goat milk was preferred for the initiation of complementary feeding; however, the cost of purchasing milk was identified as an important barrier. If caregivers failed to access milk, other liquids, such as fenugreek or cabbage fluid, sugared water, tea, soft drinks, packed juice or biscuits dissolved in water were provided. (While the type of juice was not specified by the respondents, packed juice is often flavoured with sugar.) Thin porridge made of a cereal mix flour with sugar added was also employed as a first complementary food.

Solid and semisolid foods were reported to be delayed to 9–12 months of age. The initiation of complementary feeding with liquid food was motivated by the perception that the stomach of the child has the capacity to digest these foods, while solid foods were perceived as heavy. (Shiro, a traditional porridge of barley or sorghum flour, is also reported as a food item for infants.) Moreover, participants also mentioned that liquid foods resulted in a reduced frequency of defecation, reducing the requirement for more regular cleaning of the child.

I don't give Lafiso (semi‐solid food) food until 1 year … solid food is difficult for the child who starts to feed new foods, it becomes difficult (Experienced mother, Haqabas kebele).

I choose which liquid food that can be easily swallowed for the child; after identifying the liquid and soft foods, I cook like milk, Shiro and feed the child. (Inexperienced mother, Hula Jeneta)

HEW interviewed also highlighted that they advised mothers to prepare a thin porridge using a mix of cereal and legume‐based flour to be used at the beginning of complementary feeding while delaying other semisolid and solid foods.

We provide advice about how she should feed a [child], how many times she should feed and how she should prepare a porridge … for a child who is 6 to 11 months old. (Health worker, Kortu)

In all the discussions with mothers, grandmothers and fathers, it was clear that bottle feeding was the most common method of child feeding during the introduction of complementary foods, given that primarily liquid foods are provided. Relative to other methods of feeding (spoon‐feeding or child‐initiated feeding), bottle feeding is perceived as more convenient and time‐efficient.

Both early and delayed introduction of complementary foods (inclusive of liquid foods) were reported in this sample, and the child's reported age at introduction ranged from 2 to 12 months. Early introductions were generally related to the perception that breast milk was inadequate, and the requirement that the mother separate from the infant at an early age for petty trading, attending social events such as edir (a form of social support), or engaging in domestic work, such as obtaining water and firewood.

In my case even though I don't get milk frequently I can boil tea with spices and give for my child starting from two months. (Experienced mother, Melkayee)

I spent [much time] outside petty trading. So, grandmother started [feeding the baby] at three months of age with milk and sugar. (Inexperienced mother, Hula Jeneta)

The perception of breast milk inadequacy can also arise from a shift in the mother's diet. Immediately after birth, women will generally consume a distinct diet that is heavier in more expensive animal‐source foods (meat, milk and butter). This diet may be consumed for up to 3 months; however, women in poorer households or women who begin to engage in income‐generating activities outside the household will return to a typical diet more rapidly, and this shift is often associated with the belief that breast milk is now insufficient.

By contrast, delayed introductions of complementary foods (particularly of solid food) were related to an overreliance on breast milk, and children's dislike or rejection of the first food or foods introduced, particularly if the initial foods introduced were overly spicy.

To sum up, mothers and caregivers in this context use a variety of complementary feeding practices that may not be optimal for diverse reasons. They delay the introduction of solid foods primarily for perceived health reasons (as those foods are believed to be too heavy for the digestion of infants under around a year), and also prioritise bottle and liquid feeding for convenience given maternal time constraints. Their choice of complementary foods and the associated timing is also shaped by maternal diet and by infants' reaction to early foods, once introduced.

### 3.4 Availability and access to UPF

This investigation of complementary feeding practices also highlighted that UPF play a central role in child feeding practices in this context, particularly during the introduction of complementary foods. Despite the fact that the respondents are exclusively drawn from extremely poor households characterised by a high level of food insecurity and a general reliance on subsistence food production (Gilligan et al., 2009), they spontaneously identified a range of UPF, including biscuits such as Abuwald, Hip Pop and Glucose; soft drinks (Fanta, Mirinda) and packed juices (mango and guava juice) and lipid‐based nutrient supplements. LNS are products where the majority of the energy is provided by lipids, and are generally used to treat malnutrition; the most well‐known ready‐to‐use therapeutic LNS is PlumpyNut™ (Gera et al., 2017). We categorise LNS as an ultra‐processed food given that it is manufactured using a large range of ingredients and additives that are not generally available in unprocessed foods while noting that, in this case, the manufacturing process and additives are designed with a therapeutic purpose.

The reported level of access to LNS is particularly surprising, given that it is typically provided at health centres and health posts for children diagnosed as experiencing acute malnutrition. Respondents noted availability at health centres but also reported availability in shops and marketplaces. The research team validated this availability by visiting one to two retail sites in each kebele and learned from shop owners, HEW and field staff of the NGOs working in the area that shopkeepers obtain LNS largely through informal channels. These products are diverted from the formal health system, or imported from neighbouring regions of Somalia.

Overall, respondents report a surprising level of access to UPF in conjunction with challenges in accessing other food items that would generally be considered to be more basic, including animal‐source foods and a wider variety of fruits and vegetables. Households can access subsistence food products (grain and legumes) from their own production or in local markets, but report that animal‐source foods or more diverse fruits and vegetables are unaffordable for their own consumption or can be consumed only episodically when they have access to more income. However, UPF are reported to be generally affordable (at least in small quantities) in virtually all sample areas.

### 3.5 Perceptions of UPF

UPF were identified by respondents as affordable because they are packaged in small quantities; by contrast, the cereal and legume mix flours that are used for local complementary food preparation are not available in shops or markets in similarly affordable quantities. Accordingly, families report that the purchase of the various ingredients to constitute the complementary food mix requires financial resources that they do not have. High rates of price inflation and limited opportunities for income generation are also identified as relevant challenges that may encourage households to purchase small quantities of UPF to feed infants and young children.

To start [providing] complementary foods several issues are challenging; for the additional foods, providing soft food is beneficial; however, there is lack of foods; to buy from the market the price inflation is existing; to engage in work and earn money there is no job … (Experienced mother, Kortu)

UPF were also perceived to be nutritious and effective in preventing malnutrition in children; and suitable for the early phase of complementary feeding because they can be easily diluted or dissolved in water, and thus required minimum time investment to prepare.

However, the positive perception of UPF was not uniformly shared within the sample. In particular, grandmothers generally had more negative views of these food items; such items were not widely available when they raised their own children, and thus they are more likely to be suspicious of (or simply unfamiliar with) UPF and advise their daughters or daughters‐in‐law against their use.

I advise many mothers. I have been disappointed when I heard about, we give soft drinks like FANTA. Soft drink is not important for the child. (Grandmother, Haqabas kebele)

Beyond this generational gap, there were few differences in respondents' attitudes towards UPF. Less experienced mothers were more likely to report that they sought out advice or guidance on appropriate nutritional practices from others (usually their own mothers or mothers‐in‐law), but did not as a result develop any differential perceptions of UPF.

Health workers, by contrast, reported that they advised mothers against utilising UPF, and expressed frustration that mothers were inattentive to their advice and showed a preference for commercially available foods. They interpreted mothers' preference as reflecting susceptibility to the branding of these foods as more desirable or modern, and thus having nutritional qualities superior to traditional foods.

They are interested only with foods like Fafa and plumpynut foods to be given for children … Fafa is the food that the government supplies or gives for children exposed to severe and medium malnutrition. They have such interest and don't believe with what we are telling them. They think the food we are telling them to prepare and feed their children is not balanced diet food and think only as Fafa and plumpy nut is balanced diet food. (Health Worker, Milkayee)

(Fafa is a corn soya blend flour, i.e., distributed by the government as well as nongovernmental aid agencies as food assistance during drought periods.)

### 3.6 Use of UPF

UPF are generally provided as part of a liquid diet. More specifically, to facilitate bottle‐feeding for young children, biscuits are dissolved in water, and packed juices, soft drink and LNS are reported to be diluted with water. (Given that LNS is not generally water‐soluble, it may be that the effective dilution is minimal, or mothers may not have fully or accurately reported how they incorporated LNS into a liquid diet.)

But after 6 months, [I feed] liquid foods which are found in medebir(shops) like Fanta, sprite, chocolates provided for children; liquid foods enable children not to stay in food shortages (malnourished). (Inexperienced mother, Hula Jeneta)

Mothers who receive plumpy nut for their children either from health posts or buy from shops cease preparing and giving complementary [foods] thinking that the plumpy nut is enough. (Inexperienced mother, Kortu)

Diluted ‘Glucose’ biscuits in water or sugar in the water or milk can be given. (Experienced mother, Kortu)

Caregivers primarily relied on diluted UPF for reasons of time. The convenience of providing diluted foods via bottle was a key factor identified by mothers that makes the use of these food items more attractive. Preparing the porridges recommended by health workers as complementary foods is time‐consuming (even if ingredients can be procured), and mothers may also not be able to devote time to spoon‐feeding infants given their broader domestic responsibilities, including caring for other children.

It is important to note that the dilution of UPF for young children creates a need for safe and potable water; however, scarcity of water is a challenge frequently identified by respondents when asked about the challenges of initiating complementary feeding for their children.

The main problem of this area is the problem of shortage of water; most of the time our children [are thirsty for water], scream for a long time, become tired and fall asleep; even there is no water, but we talk about balanced diet and complementary foods. (Inexperienced mother, Kortu)

## 4 DISCUSSION

Key findings from this qualitative investigation suggest that UPF are widely used as a food source for infants and young children in this study site, against a broader background of suboptimal complementary feeding practices. Both early and late initiation of complementary feeding are reported, with the provision of liquid food initiated between 2 and 12 months and the provision of semisolid and solid foods delayed until 9–12 months of age, primarily because of the perception that children's stomachs are not ready to digest it before this point. UPF (particularly lipid‐based nutrient supplements) are reported to be widely available and are used as complementary foods when mixed with water to form a liquid for bottle‐feeding or added to thin porridge as a sweetener. Participants reported that they are advised against using UPF for infants and young children by health workers as well as some community members. However, mothers continue to use these items, primarily because of time and resource constraints that render the purchase of small packages of highly processed foods feasible and appealing.

Our paper contributes the first qualitative evidence around the social, cultural and economic factors that influence of consumption of ultraprocessed foods among infants and young children in an LMIC, drawn from a socioecological model that highlights the physical, mental and social contexts surrounding infant feeding practices. Growing quantitative evidence has suggested that these consumption levels are high and growing (da Rocha et al., 2021; Nordhagen et al., 2019); in Ethiopia, one recent paper suggests that up to one in five infants and young children consumes UPF (Tizazu et al., 2022). However, this evidence does not enable us to understand the underlying perceptions and patterns of behaviour that lead parents and other caregivers to provide UPF for infants and young children.

In the realm of qualitative evidence, a larger literature has analysed factors shaping food choice (Karanja et al., 2022) and UPF consumption specifically among adolescents and adults in LMICs, though generally not in Ethiopia (Colozza, 2022; Nguyen et al., 2021; Trübswasser et al., 2021; Verstraeten et al., 2014). Evidence of rural populations is also relatively scarce, as much of the literature focuses on urban areas (Karanja et al., 2022). While this literature is diverse, key factors shaping the choice of UPF highlighted include convenience and widespread availability; these foods' relative affordability; and social desirability and shifting tastes. In Ethiopia, previous qualitative investigations related to nutrition and diet have centred around nutritional practices and taboos for adult women during pregnancy rather than examining IYCF practices, concluding that it is common for pregnant women to avoid certain foods (particularly animal‐source foods) and restrict portions (Demilew et al., 2020; Eyasu et al., 2022; Tesfa et al., 2023; Wondmeneh, 2022; Zerfu et al., 2016).

In this investigation, a range of factors drawn from the original socioecological model were found to play into parents' and caregivers' choices around providing processed foods to infants and young children, similar to those identified in previous qualitative analyses of UPF consumption outside of Ethiopia. Respondents' choices were shaped by convenience, cost and perceptions that UPF were of appropriate quality. Convenience seemed particularly salient as a criterion, given that mothers with caregiving responsibilities often face sharp time constraints. In contrast to qualitative evidence around nutritional practices for pregnant women, however, there is no evidence that taboos or the required abstention from certain food groups plays a role in IYCF feeding practices or contributes to the choice of processed foods.

The evidence presented here suggests that the child feeding practices observed pose a dual risk to optimal child growth and development. First, there is the risk of illness that might result from consuming contaminated water or complementary foods diluted with unclean water, identified as an immediate cause of malnutrition in the recent UNICEF conceptual framework (UNICEF, 2021). Previous evidence has suggested that water insecurity is a meaningful phenomenon in rural Ethiopia associated with higher stress for women (Stevenson et al., 2012), and the absence of safe water and appropriate sanitation systems is associated with a higher prevalence of child diarrhoea (Usman et al., 2019). Accordingly, the dissolution of UPF in water for bottle‐feeding in a context of widespread shortages of safe water can increase the risk of diarrhoeal disease and other forms of gastroenteritis, relative to more traditional complementary foods such as porridge that also entail boiling water. In addition, increased demand for water may increase time requirements for women to procure water given long distances to water sources (Bogale & Urgessa, 2012; Stevenson et al., 2012).

Second, the findings suggest that foods provided to infants and young children in this context are generally characterised by an inadequate nutritional profile; neither the thin porridge made of locally available foods with sugar (known as shiro or shuuroo) nor UPF mixed with water provide sufficient nutrients needed for proper growth and development (Monteiro et al., 2012). UPF, in particular, are known to be carbohydrate‐dense but poor nutritionally (Gupta et al., 2019), and may also displace more nutrient‐dense or healthier foods (Pries et al., 2019). Previous literature has suggested that consumption of UPF is associated with higher sugar content and can have adverse impacts on anthropometric profiles (Costa et al., 2019; Neri et al., 2019).

Recent evidence suggests that early childhood food experience serves as the foundation for the continuing development of food preferences across the lifespan (Ventura & Worobey, 2013), and early consumption of UPF may have long‐term health effects (Filgueiras et al., 2019; Gearhardt & Schulte, 2021). In the study site, children are exposed to sweet foods, particularly via the consumption of dissolved and diluted UPF. The sweet taste of these liquid diets is distinct from the regular cereal and legume‐based complementary foods made locally and may increase abhorrence to local foods.

This study has a number of strengths and limitations. One limitation is that data collection was conducted only with extremely poor households who are PSNP beneficiaries. Similarly, parents and caretakers who are particularly constrained in time availability (perhaps reflecting the requirement to engage in income‐generating activities) may be underrepresented due to their inability to join the focus groups. A second limitation is that the study was conducted in a single region relatively proximate to Somalia, and the ready importation of UPF (particularly LNS) across this international border may locally influence the availability of complementary foods. At the same time, one notable strength of the study is that it included interviews with diverse respondents (not only mothers but also fathers, grandmothers and health workers) to facilitate the aggregation of distinct perspectives. Moreover, the research team's composition of insiders and outsiders to the study context contributed to reflexiveness throughout the analytic process.

## 5 CONCLUSION

Evidence from this formative qualitative study suggests that UPF are widely employed as complementary food for young children in rural Oromia, particularly because they are perceived by caretakers as convenient and ready to use. Moreover, in the context of widespread price inflation of the milk, legumes and cereals that are traditionally used as ingredients for preparing nutritious local complementary foods, the availability of UPF (including LNS reportedly diverted from the formal health system) sold in small packages with a minimum affordable price is appealing to households that are income‐constrained. Further research is required to quantify the level of consumption of UPF by children and determine the health, nutritional and dietary effects of the pattern currently observed. Properly designed nutritional education targeting a range of caretakers (including fathers and grandmothers) is required to address the broader pattern of suboptimal complementary feeding practices (the delayed introduction of semisolid and solid foods to young children) and the overuse of UPF. Deploying other intersectoral or livelihood‐focused interventions (with a focus on enhancing the variety of food groups available from home production) may also be beneficial in the context of wide price inflation.

## AUTHOR CONTRIBUTIONS

Jessica Leight conceived of the research. Elazar Tadesse and Jessica Leight designed the study. Elazar Tadesse coordinated the field work and supervised the data collection. Ibrahim Abdirahman, Shiferaw Letta and Elazar Tadesse collected data. Elazar Tadesse conducted data analysis with the support of Ibrahim Abdirahman and Shiferaw Letta. Elazar Tadesse and Jessica Leight wrote the manuscript, and all authors reviewed the final manuscript.

## CONFLICT OF INTEREST STATEMENT

The authors declare no conflict of interest.

## Data Availability

The data that support the findings of this study are available from the corresponding author upon reasonable request.
